# Non-coding RNA: a new perspective on the regulation of secondary metabolites in medicinal plants

**DOI:** 10.1186/s43897-026-00259-2

**Published:** 2026-07-04

**Authors:** Shuaibiao Zhang, Susu Chen, Lei Zhang

**Affiliations:** 1 School of Pharmacy, Shanghai University of Traditional Chinese Medicine, Shanghai, 201203 China; 2 Department of Pharmaceutical Botany, School of Pharmacy, Naval Medical University, Shanghai, 200433 China; 3 State Key Laboratory for Quality Ensurance and Sustainable Use of Dao-Di Herbs, Beijing, 100700 P. R. China

**Keywords:** Non-coding RNA, Medicinal plant, Secondary metabolism

## Abstract

Medicinal plants are a valuable reservoir of diverse secondary metabolites (SMs), which serve as essential sources for natural drug development. However, the natural production of these bioactive compounds is generally low. Moreover, the biosynthetic processes of these SMs are regulated by a sophisticated network involving transcription factors, post-translational modifications, as well as plant hormones and environmental factors. Recent studies have identified an increasing number of non-coding RNAs (ncRNAs) and confirmed their roles in regulating SM biosynthesis. In this review, we systematically summarize the research progress on the regulation of SM biosynthesis by ncRNAs in medicinal plants, including microRNAs (miRNAs), long non-coding RNAs (lncRNAs), circular RNAs (circRNAs), and phased secondary siRNAs (phasiRNAs). Additionally, we summarize ncRNA‑mediated studies in staple crops, horticultural plants, and woody species to provide references for medicinal plant research. Finally, we discuss future research directions for ncRNAs in medicinal plants, including the construction of spatiotemporal regulatory networks, the application of artificial intelligence (AI)-assisted approaches, the elucidation of molecular regulatory mechanisms, and potential applications of ncRNAs in the standardized production of medicinal plants.

## Introduction

Medicinal plants, which are botanical species known for their therapeutic properties, play a crucial role in the prevention and treatment of diseases (Li et al. 2025c). These plants synthesize and accumulate SMs in various organs, including roots, stems, leaves, flowers, fruits, and seeds. Although SMs do not participate directly in primary metabolism, they are vital for the plant’s adaptation to environmental fluctuations and significantly enhance resistance to biotic and abiotic stresses. SMs in medicinal plants are primarily categorized into three groups based on their chemical structures and biosynthetic pathways: phenolics, terpenoids, and alkaloids. These compounds, once identified and extracted, not only serve as valuable resources for novel drug development but also have applications in various industries such as food, fragrance, and cosmetics (Richard et al. 2024, Li et al. 2025c).

Currently, three main strategies are utilized to obtain SMs: chemical synthesis, microbial biosynthesis, and direct extraction from plants. Chemical synthesis, not constrained by the availability of plant resources, can produce target compounds in higher yields and within a shorter duration. However, this method often results in significant material loss, and the catalysts and by-products generated may contribute to environmental pollution. Microbial biosynthesis employs engineered chassis cells, such as *Saccharomyces cerevisiae* and *Escherichia coli*, to convert feedstocks into various compounds through fermentative pathways. Despite this, most compounds synthesized via microbial cell factories remain in preliminary stages of research, with only a few reaching industrial-scale production (Jay et al. 2012). A prominent example is the production of artemisinin, where artemisinic acid is synthesized in yeast and subsequently converted into semisynthetic artemisinin through chemical processes (Turconi et al. 2014, Paddon et al. 2013). However, the high production costs of this method pose significant challenges to its industrial-scale application (Mark 2016). Consequently, direct extraction from plants continues to be the predominant method for obtaining the majority of SMs.

**Fig. 1:**
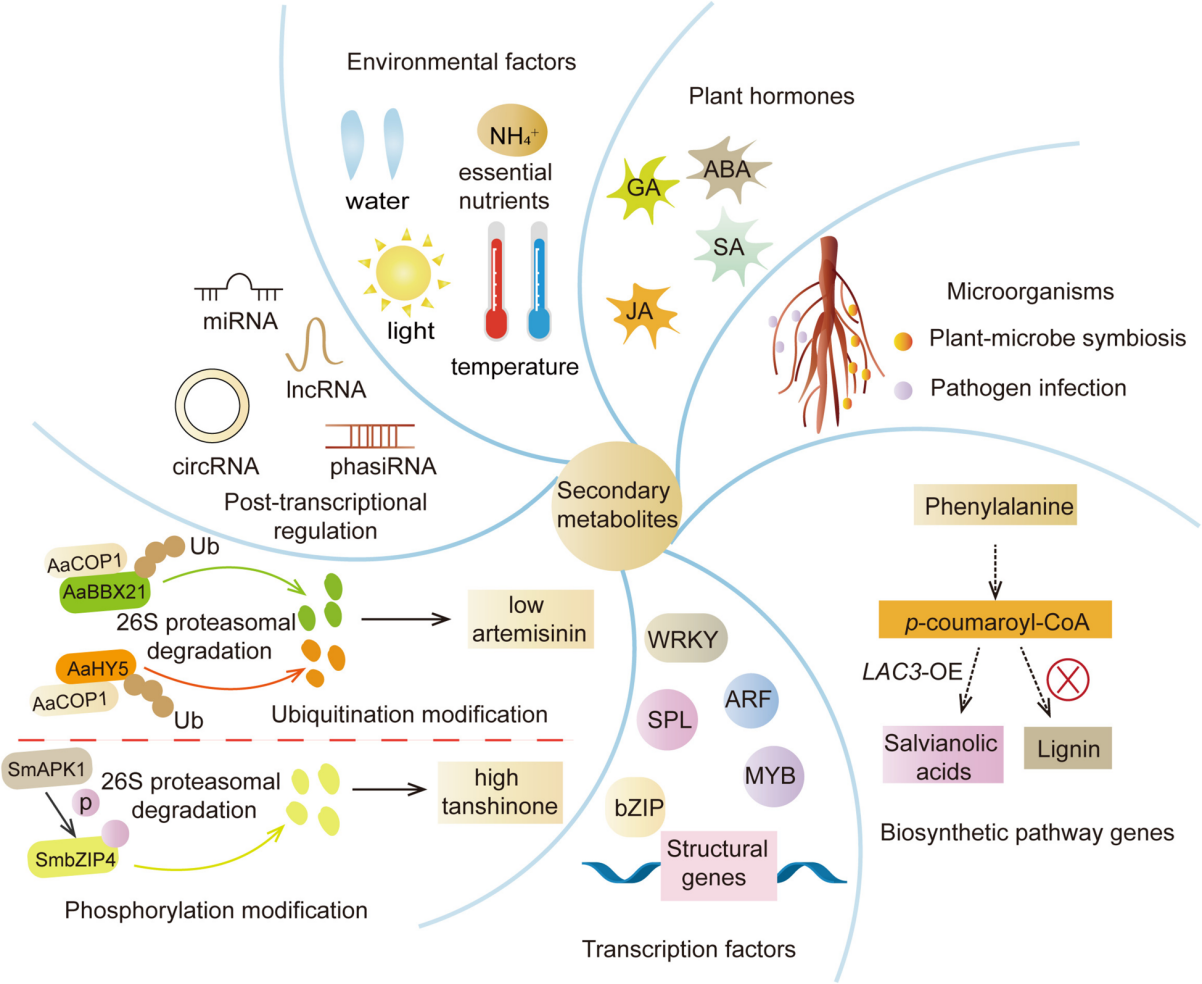
Strategies for regulating SMs biosynthesis in medicinal plants. These strategies encompass both external and internal regulatory mechanisms. External factors include environmental conditions (e.g., water, light, temperature, and nutrient availability), phytohormone treatments, and interactions with microorganisms or endophytes. Internal regulatory mechanisms involve the manipulation of biosynthetic pathway genes, TFs, post-translational modifications, and ncRNAs. In *Salvia miltiorrhiza (S. miltiorrhiza)*, *p*-coumaroyl-CoA serves as the common precursor for both salvianolic acids and lignin biosynthesis. Laccase (*SmLAC3*) a pathway enzyme gene involved in salvianolic acid biosynthesis, plays a pivotal role in directing metabolic flux. Overexpression of *SmLAC3* in *S. miltiorrhiza* has been shown to significantly increase the accumulation of salvianolic acids in roots while concurrently reducing lignin content, suggesting a redirection of precursors from the lignin pathway toward salvianolic acid biosynthesis (Zou et al. 2021). In *A. annua*, the BBX domain-containing protein (AaBBX21)-elongated hypocotyl 5 (AaHY5) complex positively regulates artemisinin biosynthesis. Constitutively photomorphogenic 1 (AaCOP1), an E3 ubiquitin ligase involved in light signal transduction, functions prominently under dark conditions. AaCOP1 ubiquitinates and promotes the degradation of both AaBBX21 and AaHY5. This degradation attenuates their transcriptional activation of downstream target genes, including glandular trichome-specific WRKY 1 (*AaGSW1*), MYB family protein 108 (*AaMYB108*), and APETALA2/ethylene-response factor family TF (*AaORA*). Consequently, the downregulation of these biosynthetic regulators ultimately leads to reduced artemisinin accumulation (He et al. 2024a). The basic region leucine zipper (bZIP) family TF, SmbZIP4, acts as a negative regulator of tanshinone biosynthesis in *S. miltiorrhiza*. Conversely, SmAPK1, a protein kinase, functions as a positive regulator. Mechanistically, SmAPK1 phosphorylates SmbZIP4 at Ser97 and Thr99, which promotes the degradation of SmbZIP4 via the 26S proteasome. The resulting removal of this transcriptional repressor ultimately causes a significant increase in tanshinone accumulation (Zhu et al. 2024b). Ub, ubiquitin; p, phosphorylation

The yield of SMs is primarily constrained by their abundance, which is influenced by numerous factors, including the plant genotype, developmental stage, growth year, and organ type. However, medicinal plants typically exhibit low concentrations of SMs. For instance, the concentration of artemisinin in *Artemisia annua* (*A. annua*) constitutes less than 1% of its dry weight (Zhao et al. 2024), and the paclitaxel content in the bark of the *Taxus* species is approximately 0.04% (Jiang et al. 2024a). In response to the increasing global demand, researchers have developed various strategies to enhance the content of SMs in medicinal plants (Fig. 1).

The accumulation of SMs can be significantly influenced by environmental factors such as water availability, light intensity, temperature, and mineral nutrition (Li et al. 2020). Phytohormones serve as crucial elicitors that govern essential physiological processes in plants, including growth, development, and senescence. The exogenous application of hormones, such as abscisic acid (ABA) and methyl jasmonate (MeJA), has been shown to affect the biosynthesis of SMs (Li et al. 2025b). Furthermore, certain microorganisms and endophytes enhance the accumulation of SMs through mechanisms including nitrogen fixation and hormone synthesis (Yu et al. 2024, Chen et al. 2025a). At the molecular level, the biosynthesis of SMs is directly influenced by the expression of genes associated with biosynthetic pathways. The overexpression of genes within synthetic pathways or the suppression of those in branch pathways can redirect metabolic flux, thereby enhancing the conversion of precursor substances into desired products (Li et al. 2024a). Transcription factors (TFs) play a pivotal role in the regulation of gene expression by binding to cis-elements within promoter sequences, integrating upstream signaling cascades, and modulating the expression of biosynthetic genes, either activating or repressing them (Yang et al. 2012). Beyond transcriptional control, post-translational modifications, notably phosphorylation and ubiquitination, also influence the biosynthesis of SMs (Liu et al. 2023b, Zhu et al. 2024b, He et al. 2024b).

**Fig. 2:**
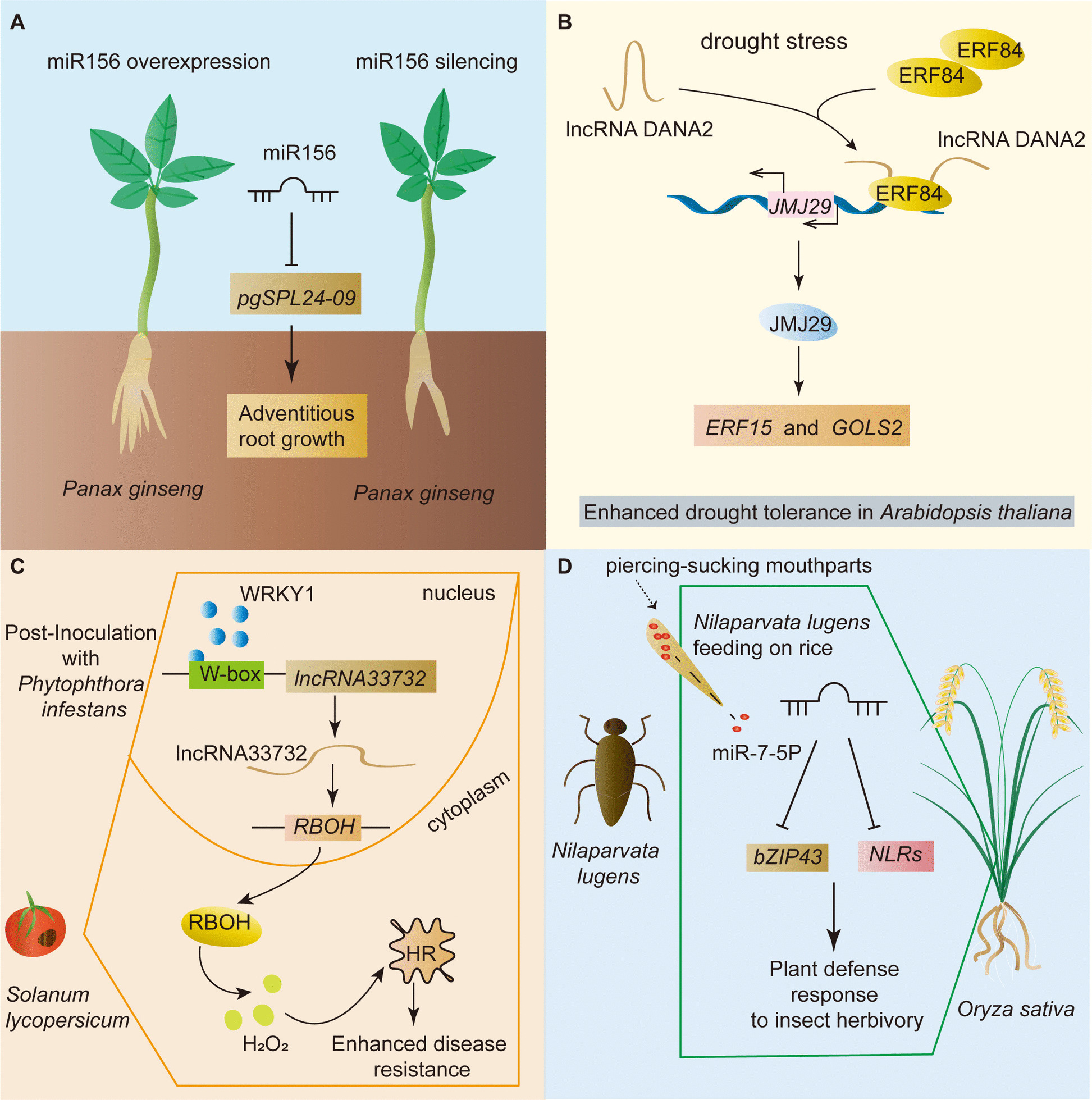
The multifaceted regulatory roles of ncRNAs in plants. **A**. miR156 regulates the formation of adventitious roots in *Panax ginseng*. Overexpression of miR156 significantly increases the number of adventitious roots, whereas inhibition of miR156 reduces their number (Jiang et al. 2024b). **B**. lncRNA DANA2 enhances drought resistance. Under drought stress conditions, lncRNA DANA2 recruits ERF84 to jointly promote the expression of the histone demethylase gene *JMJ29*, which subsequently facilitates the transcriptional upregulation of ethylene response factor 15 (*ERF15*) and galactinol synthase2 gene (*GOLS2*), both established as positive regulators of drought stress responses (Zhang et al. 2023a). **C**. lncRNA33732 enhances disease resistance. Following infection by *Phytophthora infestans*, WRKY1 binds to W-Box elements within the promoter sequence of *lncRNA33732*, activating its expression. This, in turn, promotes the expression of the *RBOH* gene, leading to increased accumulation of H₂O₂. This H₂O₂ accumulation contributes to enhanced disease resistance against the pathogen (Cui et al. 2019). **D**. Cross-kingdom regulation: brown planthopper miR-7-5p suppresses rice insect resistance. During feeding, brown planthopper secretes miR-7-5p into rice tissues through its mouthparts. This miR-7-5p suppresses the expression of the insect-resistant gene basic Leucine Zipper 43 (*bZIP43*), thereby weakening the plant’s insect resistance (Zhang et al. 2024)

ncRNAs play crucial roles in a variety of cellular and physiological processes. They do not encode proteins and are broadly categorized into housekeeping and regulatory types. Housekeeping ncRNAs encompass transfer RNAs (tRNAs), ribosomal RNAs (rRNAs), small nuclear RNAs (snRNAs), and small nucleolar RNAs (snoRNAs). Regulatory ncRNAs are primarily divided into miRNAs, lncRNAs, circRNAs, and short interfering RNAs (siRNAs) (Kiriga et al. 2020). In *Panax ginseng*, miR156 precisely controls the growth of adventitious roots (Fig. 2A) (Jiang et al. 2024b). Under drought stress conditions, the lncRNA DANA2 forms a regulatory complex with ethylene response factor 84 (ERF84), subsequently activating the expression of the JmjC domain-containing protein 29 (*JMJ29*), which influences drought adaptation in *Arabidopsis thaliana* (*A. thaliana*) (Fig. 2B) (Zhang et al. 2023a). In *Solanum lycopersicum* (tomato), the lncRNA33732, activated by WRKY family protein 1 (WRKY1), induces the expression of the respiratory burst oxidase homolog (*RBOH*) gene and increases hydrogen peroxide (H₂O₂) accumulation, thereby enhancing resistance to *Phytophthora infestans* infection (Fig. 2C) (Cui et al. 2019). Notably, ncRNAs possess cross-kingdom regulatory capacities that facilitate interspecies communication between plants and other organisms. For example, when *Nilaparvata lugens* (brown planthopper) feeds on *Oryza sativa* (rice), miR-7-5P is secreted into the rice and suppresses the expression of insect-resistant genes in the host plant (Fig. 2D) (Zhang et al. 2024). Given the significant functions of ncRNAs in plants, a systematic review focused on the regulatory mechanisms of ncRNAs in SM biosynthesis is warranted. This review discusses recent research on ncRNA-mediated regulation of SM biosynthesis in medicinal plants. Furthermore, we also review ncRNA-mediated studies in other species and propose future research directions.

### Brief introduction to miRNA

miRNAs constitute a class of small, approximately 21-nucleotide-long, single-stranded, ncRNAs that are ubiquitously present in eukaryotic cells. The biogenesis of miRNAs and their mechanisms of action exhibit significant differences between animals and plants (Achkar et al. 2016). In animals, the biogenesis of miRNAs involves the specialized endonuclease Drosha, which performs the initial cleavage of primary miRNA transcripts (pri-miRNAs) within the nucleus. The resulting precursor miRNAs (pre-miRNAs) are then exported to the cytoplasm, where the enzyme Dicer executes the secondary cleavage. In contrast, the processing of miRNAs in plants occurs entirely within the nucleus, where RNA polymerase II (Pol II) transcribes miRNA genes (*MIR*) to yield pri-miRNAs. These are subsequently processed by Dicer-Like 1 (DCL1) into pre-miRNAs. With the assistance of Hyponastic Leaves 1 (HYL1) and serrate (SE), DCL1 further processes pre-miRNAs to produce double-stranded miRNA duplexes. These duplexes undergo methylation mediated by hua enhancer 1 (HEN1). Subsequently, one strand of the duplex is incorporated into the RNA-induced silencing complex (RISC) through association with argonaute (AGO) proteins (Yu et al. 2017, Zhan et al. 2022).

### miRNAs-mediated regulation of SMs biosynthesis

miRNAs play a pivotal role in gene regulatory networks of plants (Tang et al. 2017, Marco et al. 2017). Typically, the base-pairing between a miRNA and its target mRNA is near-perfect, with miRNAs predominantly mediating gene silencing through targeted mRNA cleavage (Wang et al. 2019). The advent of genome sequencing, transcriptome sequencing (RNA-seq), and small RNA sequencing (sRNA-seq) has greatly facilitated the functional characterization of miRNAs in medicinal plants. These miRNAs primarily exert their function through two mechanistic approaches: targeting TFs that govern metabolic pathways, and regulating genes encoding biosynthetic enzymes. For analytical clarity, we categorize the SMs regulated by miRNAs into several classes, including phenolics, terpenoids, and alkaloids.

### miRNAs involved in phenols biosynthesis

Phenolic compounds manifest a wide range of pharmacological activities, including antibacterial, antioxidant, and anti-inflammatory properties (Zhao et al. 2023). Their biosynthesis predominantly takes place via the phenylpropanoid pathway, with L-phenylalanine serving as the initial substrate. Phenylalanine ammonia-lyase (PAL), the pathway’s initial key enzyme, catalyzes the deamination of L-phenylalanine to trans-cinnamic acid. This is followed by a series of modification reactions involving cinnamic acid, such as hydroxylation and methylation, culminating in the formation of *p*-coumaroyl-CoA. This compound acts as the central intermediate in the biosynthesis of most phenolic compounds. The pathway then diverges, leading to the production of various phenolic compounds (Deng et al. 2024).

**Fig. 3:**
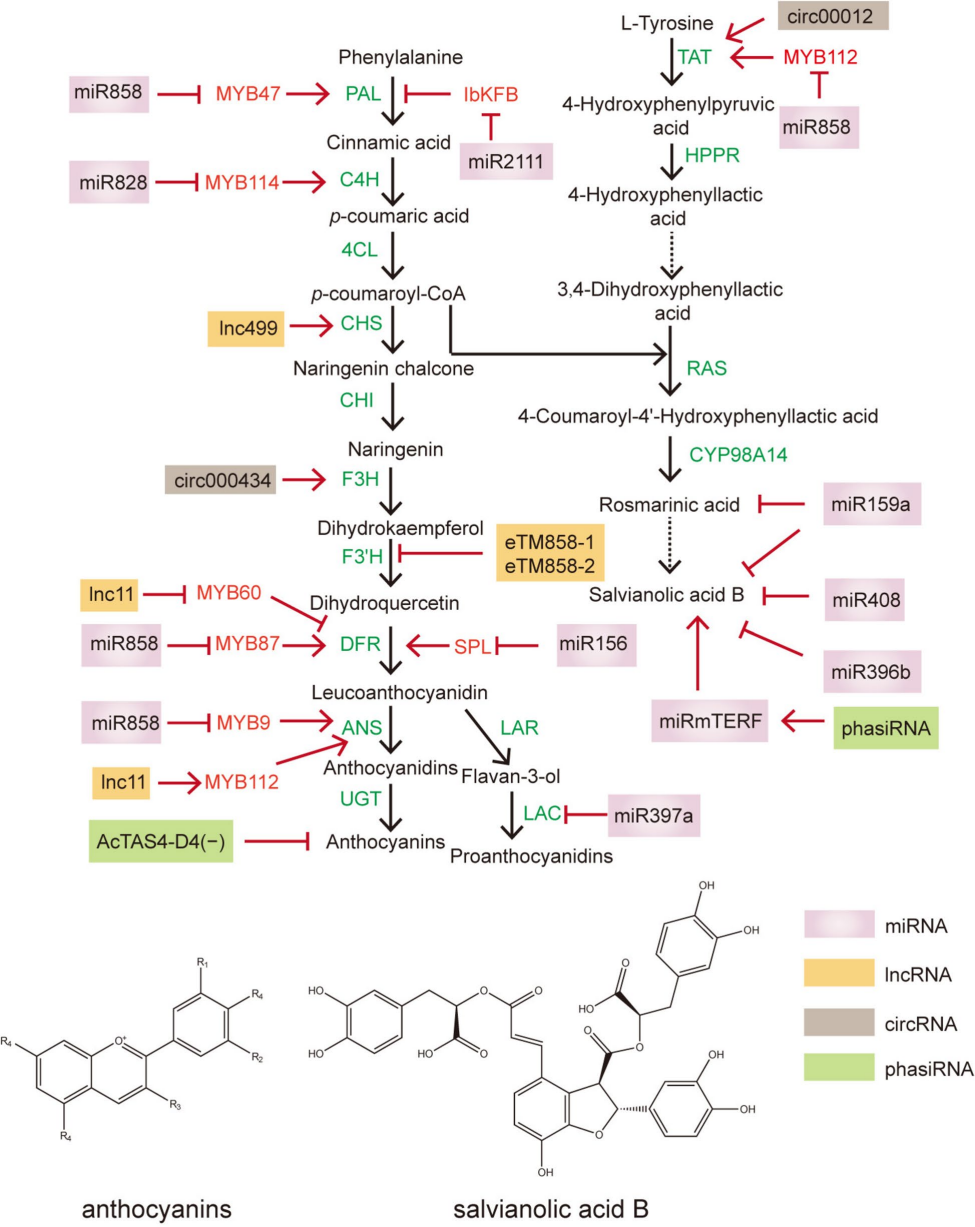
Regulatory network of ncRNAs in phenolic compound biosynthesis. This schematic diagram elucidates the regulatory roles of ncRNAs in the biosynthesis of phenolic compounds, focusing on the biosynthetic pathways of anthocyanins and salvianolic acid B as representative models. It summarizes known miRNAs that target essential structural genes or TFs in phenolic metabolism across various plant species. Additionally, the diagram includes lncRNAs, circRNAs, and phasiRNAs, which have been identified as potential regulators of phenolic compound biosynthesis, thereby underscoring the intricate complexity of ncRNA-mediated metabolic regulation. Green text indicates structural genes and red text indicates TFs or modifying enzyme. C4H, cinnamic acid 4-hydroxylase; F3H, flavanone 3-hydroxylase; ANS, anthocyanidin synthase; HPPR, 4-hydroxyphenylpyruvate reductase; RAS, rosmarinic acid synthase; CYP98A14, cytochrome P450 98A14; 4CL, *p*-coumaroyl coenzyme A ligase; CHI, chalcone isomerase; UGT, UDP-glycosyltransferase; LAR, leucoanthocyanidin reductase

Phenolic acids, primarily the water-soluble bioactive components in *S. miltiorrhiza* (e.g., salvianolic acids A and B), are extensively employed in treating cardiovascular and cerebrovascular diseases (Li et al. 2018). miRNAs dynamically regulate the biosynthesis of phenolic acids (Fig. 3). For instance, overexpression of miR159a significantly decreases the levels of rosmarinic acid and salvianolic acid B (Zhou et al. 2024), miR408 directly inhibits the expression of *SmLAC3*, thereby reducing the accumulation of salvianolic acid B (Zou et al. 2021). Conversely, the miR858a-*SmMYB6/SmMYB112* module enhances the expression of *PAL* and tyrosine aminotransferase (*TAT*), thereby promoting the synthesis of phenolic acids (Zhu et al. 2024a). Notably, the same miRNAs can have opposing effects on distinct metabolic branches within the same species. Overexpression of miR396b decreases the accumulation of salvianolic acid while simultaneously increasing tanshinone levels (Zheng et al. 2020). Meanwhile, miR397a-mediated downregulation of the *LAC* genes redirects metabolic flux from proanthocyanidin biosynthesis toward the production of catechins and epicatechins, demonstrating miRNA-directed flexibility in SMs partitioning (Li et al. 2025a, 2025a). In *Camellia sinensis* (*C. sinensis*), young leaves and apical buds accumulate abundant SMs with significant health benefits. *CsTCP3* and *CsTCP4* are part of the teosinte branched 1 (TB1)/cycloidea (CYC)/proliferating cell nuclear antigen factor 1 (PCF1) (TCP) family. miR319 modulates catechin production by directly targeting *CsTCP3* and *CsTCP4* (Yu et al. 2021). Additionally, miR156 suppresses the expression of its target gene squamosa promoter-binding-like (*SPL*), indirectly influencing the expression of dihydroflavonol 4-reductase (*DFR*), a critical enzyme in the catechin biosynthetic pathway (Fan et al. 2015).

Flavonoids, distinguished by their core C6-C3-C6 skeleton, constitute a significant subclass of phenolic compounds. The biosynthetic process begins with chalcone synthase (CHS), which catalyzes the condensation of one molecule of *p*-coumaroyl-CoA with three molecules of malonyl-CoA to produce naringenin chalcone. Chalcone isomerase (CHI) then catalyzes the stereospecific isomerization of naringenin chalcone to naringenin, the pivotal intermediate in flavonoid biosynthesis. Naringenin may subsequently be transformed into various flavonoid classes, including flavones, isoflavones, dihydroflavonols, and anthocyanins, through multiple enzymatic steps (Leonte et al. 2023). In *Curcuma longa* (turmeric), miR2919 regulates flavonoid biosynthesis by modulating the expression of the flavanone synthase gene (Singh et al. 2017). Under cold stress, miR858 may influence flavonoid accumulation in *Astragalus Membranaceus* by targeting and regulating MYB TFs within the phenylpropanoid pathway (Abla et al. 2019). Furthermore, following short-term UV-B radiation exposure in *Chrysanthemum morifolium* (chrysanthemum), the expression of several miRNAs related to flavonoid biosynthesis was found to be suppressed (Yang et al. 2020). In *Scutellaria baicalensis*, miR858 suppresses the expression of *SbMYB47*, a TF that activates the expression of phenylalanine ammonia-lyase 3 (*SbPAL-3*) and flavone synthase II (*SbFNSⅡ−2*), thereby modulating flavone accumulation (Yang et al. 2024).

Anthocyanins, a subgroup of flavonoids, are widely utilized as natural food colorants for their ability to impart a range of colors. Beyond their function as colorants, anthocyanins exhibit potent free radicals scavenging capacity and significant antioxidant activity (Ayvaz et al. 2022). In *Ammopiptanthus nanus*, osmotic stress activates the miR858-*MYB87* regulatory module, which modulates anthocyanin accumulation through precise regulation of the *DFR* gene expression (Sumbur et al. 2023). In *Malus domestica*, miR858 regulates fruit pigmentation by targeting critical TFs, *MdMYB9* and PA1-type MYB TF 1 (*MdMYBPA1*) (Li et al. 2023b). Proanthocyanidins and anthocyanins both derive from the enzymatic action of DFR, which converts dihydroflavonols into leucoanthocyanidins (Cao et al. 2024b). In apples, miR858 negatively affects proanthocyanidin accumulation in fruit peel by targeting *MdMYB9*, *MdMYB11*, and *MdMYB12*. Significantly, MdBBX22 binds to the *MIR858* promoter and induces its expression, thereby inhibiting proanthocyanidin accumulation and indirectly enhancing anthocyanin biosynthesis in the peel. This finding serves as a basis for further exploration into the regulatory mechanisms governing proanthocyanidin accumulation and the metabolic relationship between proanthocyanidins and anthocyanins (Zhang et al. 2022). Moreover, under light-induced conditions, miR7125 and its target gene cinnamoyl-CoA reductase (*CCR*) modulate the balance between anthocyanin and lignin biosynthesis (Hu et al. 2021).

### miRNAs involved in terpenoids biosynthesis

Terpenoids constitute a class of natural compounds synthesized via the mevalonate (MVA) pathway and the methylerythritol phosphate (MEP) pathway. These compounds are structurally characterized by their isoprene-based skeletons and are classified based on the number of isoprene units into monoterpenoids, sesquiterpenoids, diterpenoids, triterpenoids, and their derivatives. Notable examples of terpenoid compounds with established therapeutic significance include tanshinone, paclitaxel, and artemisinin (Wang et al. 2023b).

**Fig. 4:**
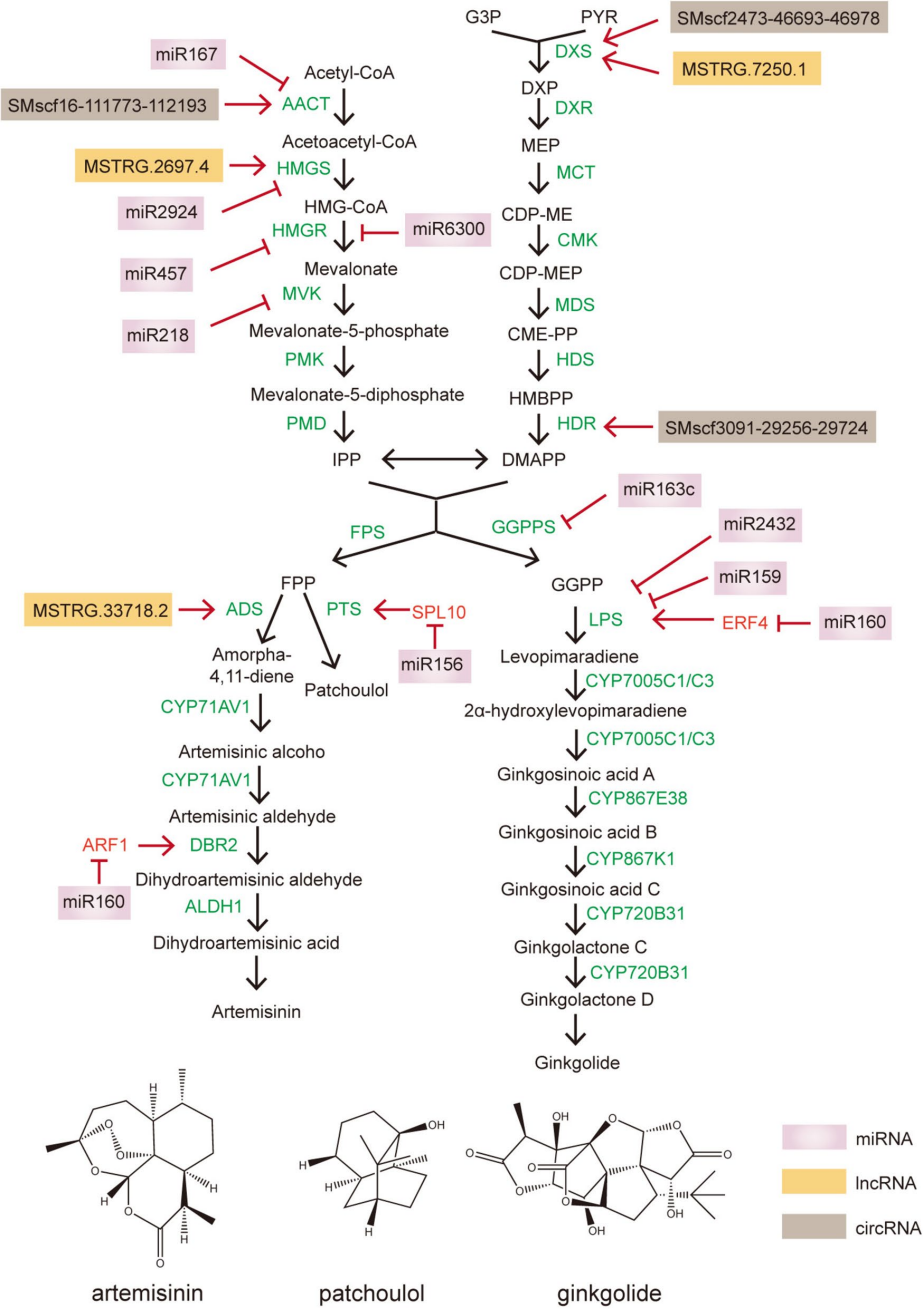
Regulatory roles of ncRNAs in terpenoid biosynthesis. This figure delineates the biosynthetic pathways of the terpenoid compounds artemisinin, patchoulol, and ginkgolide. In *A. annua*, miR160 regulates the biosynthesis of artemisinin by targeting the auxin response factor *ARF1*. In *P. cablin*, miR156 controls the biosynthesis of patchoulol by targeting *SPL10*. In *G. biloba*, miR160 influences ginkgolide biosynthesis by targeting *ERF4*. Beyond these validated instances, the figure also depicts computationally predicted miRNAs, lncRNAs, and circRNAs that may play roles in the regulation of terpenoid biosynthesis. Green text indicates structural genes and red text indicates TFs. AACT, acetoacetyl CoA thiolase; MVK, mevalonate kinase; HDR, (E)−4-hydroxy-3-methylbut-2-enyl diphosphate reductase; FPS, farnesyl diphosphate synthase; GGPPS, geranylgeranyl diphosphate synthase; LPS, levopimaradiene synthase; PMK, phosphomevalonate kinase; PMD, mevalonate diphosphate decarboxylase; DXR, 1-deoxy-D-xylulose 5-phosphate reductoisomerase; MCT, 2-C-methyl-D-erythritol 4-phosphate cytidylyltransferase; CMK, CDP-ME kinase; MDS, 2-C-methyl-D-erythritol 2,4-cyclodiphosphate synthase; HDS, (E)−4-hydroxy-3-methylbut-2-enyl diphosphate synthase; CYP71AV1, cytochrome P450 mono-oxygenase; ALDH1, aldehyde dehydrogenase 1; CYP7005C1/C3, cytochrome P450 7005C1/C3; CYP867E38, cytochrome P450 867E38; CYP867K1, cytochrome P450 867K1; CYP720B31, cytochrome P450 720B31

In the study of *Ginkgo biloba* (*G. biloba*), a systematic screening of miRNAs involved in the biosynthesis of terpene trilactones (TTLs) revealed 25 candidate miRNAs that potentially regulate TTL accumulation (Ye et al. 2020). Subsequent functional analyses showed that gb-miR160 negatively regulates the expression of *GbERF4*, thereby influencing TTL biosynthesis (Fig. 4) (Zheng et al. 2024). In *S. miltiorrhiza*, miR858a indirectly contributes to tanshinone biosynthesis by regulating the expression of the *SmMYB* gene. This gene activates the expression of two crucial biosynthetic enzymes, copalyl diphosphate synthase 1 (*SmCPS1*) and kaurene synthase-like 1 (*SmKSL1*), both of which are essential for tanshinone production (Zhu et al. 2024a). Additionally, overexpression of miR396b significantly enhances tanshinone content, with total tanshinone levels reaching 1.91-fold higher than those in wild-type plants (Zheng et al. 2020). In the paclitaxel biosynthetic pathway, two critical enzymes, taxane 13α-hydroxylase and taxane 2α-O-benzoyltransferase, have been identified as targets of miR164 and miR171, respectively (Hao et al. 2012). Studies conducted in Taxus cell lines have demonstrated that overexpression of miR8154 and miR5298b promotes the expression of genes associated with paclitaxel biosynthesis (Zhang et al. 2015). Furthermore, the integration of laser capture microdissection (LCM) with liquid chromatography-tandem mass spectrometry (LC–MS/MS)-based data-dependent acquisition (DDA) has elucidated that several enzymes involved in paclitaxel biosynthesis are predominantly localized in the endodermis. Subsequent research identified an endodermis-specific MYB1-like (MYB1L) protein and proposed a regulatory role for the miR858b-*MYB1L* module in paclitaxel metabolism (Yu et al. 2025). In *Pogostemon cablin* (*P. cablin*), the miR156-*SPL10* module regulates the biosynthesis of sesquiterpenes by modulating the expression of patchoulol synthase (*PatPTS*) (Yu et al. 2015). In *A. annua*, glandular secretory trichomes serve as the primary sites for the production and storage of artemisinin. The miR160-auxin response factor 1 (*ARF1*) module enhances artemisinin biosynthesis by regulating trichome development and activating the expression of artemisinic aldehyde Δ11(13) double bond reductase 2 (*AaDBR2*) (Guo et al. 2022b). Notably, the treatment of *A. annua* seedlings with graphene promotes their growth and increases the density of glandular trichomes by approximately 80%. Further studies have revealed that graphene inhibits miR828 expression, leading to the upregulation of its target gene *AaMYB17*, which influences trichome density and artemisinin accumulation. This discovery offers a novel approach for enhancing the yields of SMs in medicinal plants (Cao et al. 2023).

### miRNAs involved in alkaloids biosynthesis

Alkaloids constitute a diverse and structurally intricate class of nitrogen-containing SMs, with more than ten thousand distinct compounds identified so far. Their biosynthesis primarily originates from amino acid and isoprene metabolic pathways. Depending on their chemical structures, alkaloids can be categorized into several major groups, such as pyridine, isoquinoline, tropane, and indole alkaloids. Compounds such as aconitine, ephedrine, and vinblastine are chiefly known for their pharmacological effects, which include cough suppression, anticancer activity, and analgesic properties (Guo et al. 2022a).

**Fig. 5:**
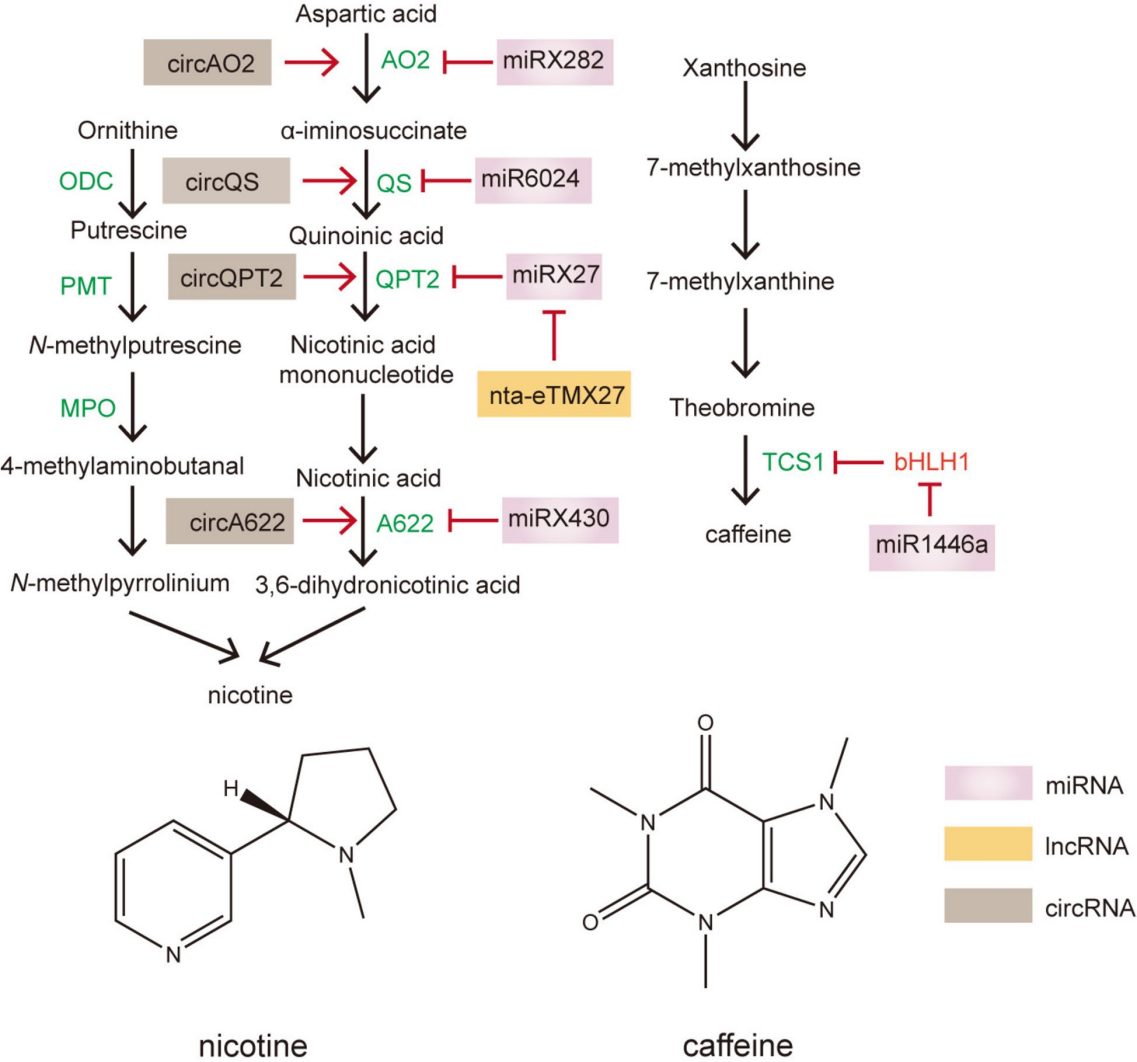
Regulatory roles of ncRNAs in alkaloid biosynthesis. This figure illustrates the biosynthetic pathways of the alkaloid compounds nicotine and caffeine. In tobacco, miRX27 regulates nicotine biosynthesis by inhibiting *QPT2* expression. In *C. sinensis*, miR1446a indirectly influences caffeine biosynthesis by suppressing *bHLH1* expression. Apart from these two cases, the other ncRNAs depicted in the figure are merely predicted and have not been experimentally validated. Green text indicates structural genes and red text indicates TFs. A622, Isoflavone reductase-like protein; TCS1, theobromine synthase 1; ODC, orn decarboxylase; PMT, putrescine methyltransferase; MPO, *N*-methylputrescine oxidase

In seedlings of *Catharanthus roseus* (*C. roseus*), researchers identified 181 conserved miRNAs and 173 novel miRNAs. Predictive analyses suggested that miR160 potentially targets several auxin response factors, specifically *CrARF10*, *CrARF16*, and *CrARF17*. Treatment with MeJA significantly increases the expression of miR160, while simultaneously decreasing the levels of *CrARF10*, *CrARF16*, and *CrARF17*. Notably, CrARF16 acts as a transcriptional repressor of critical genes in the terpenoid indole alkaloid (TIA) biosynthesis pathway, including tryptophan decarboxylase (*TDC*), strictosidine synthase (*STR*), and geraniol 10-hydroxylase (*G10H*). The suppression of *CrARF16* by miR160 relieves this repression, thereby activating TIA pathway genes and enhancing alkaloid accumulation in *C. roseus* (Ethan et al. 2017). Nicotine, a distinct alkaloid found in *Nicotiana tabacum* (tobacco), is synthesized through a pathway involving four miRNAs that target genes responsible for nicotine biosynthesis. Among these, nta-miRX27 prominently affects nicotine accumulation by modulating the expression of quinolinate phosphoribosyltransferase 2 (*QPT2*) (Fig. 5) (Li et al. 2015). Nicotine synthesis occurs exclusively in the roots of tobacco plants, with subsequent xylem-mediated transport to the aerial parts. Following topping treatment, both the number of lateral roots and the nicotine content increase in tobacco roots. Concurrently, miR164 expression decreases, whereas the expression of its target, the NAC TF *NtNAC-R1*, increase. NtNAC-R1 enhances auxin signaling, which promotes lateral root development, indicating that miR164 may negatively regulate nicotine biosynthesis (Fu et al. 2013). In *Papaver somniferum*, researchers have identified 316 conserved miRNAs and 11 novel miRNAs. Among these, miR13, miR2161, and miR408 are predicted to be involved in the biosynthesis of benzylisoquinoline alkaloids (BIA) (Hatice et al. 2015). In *C. sinensis*, miR1446a enhances caffeine biosynthesis by directly suppressing the expression of the negative regulator basic helix-loop-helix (*CsbHLH1*) (Jin et al. 2024). In *Aconitum vilmorinianum*, miR6300 is hypothesized to influence terpenoid backbone formation and regulate the synthesis of diterpenoid alkaloids by targeting hydroxymethylglutaryl-CoA reductase (*HMGR*), a key enzyme in the mevalonate pathway (Zhao et al. 2025b).

### Brief introduction to lncRNA

lncRNAs are typically defined as ncRNA transcripts that exceed 200 nucleotides in length (John et al. 2023). Based on their genomic origins, lncRNAs can be broadly categorized into three types: long intergenic ncRNAs (lincRNAs), intronic ncRNAs (incRNAs), and natural antisense transcripts (NATs), which are transcribed from the complementary DNA strands of associated genes (Wang et al. 2022). Initially, lncRNAs were frequently dismissed as transcriptional noise due to their characteristically low sequence conservation, minimal expression levels, and poor detectability in conventional genetic screens (Kyle et al. 2023). However, the advent of high-throughput sequencing technologies has facilitated the comprehensive identification of lncRNAs, leading to significant advancements in this field. Functionally, most plant lncRNAs are localized within the nucleus, where they play crucial roles in complex gene regulation processes (Chen et al. 2024). At the pre-transcriptional level, lncRNAs may regulate gene expression through the formation of chromatin loops, recruitment of epigenetic modifiers, or interactions with promoter regions. Additionally, during co-transcriptional and post-transcriptional stages, lncRNAs influence mRNA dynamics through mechanisms such as alternative splicing, enhancement of transcript stability by acting as miRNA decoys, and regulation of translation (Andrzej et al. 2021, Kyle et al. 2023).

### lncRNAs-mediated regulation of SMs biosynthesis

Cytoplasmic-localized lncRNAs regulate gene expression at the post-transcriptional level through various mechanisms, such as engaging with miRNA-mediated regulatory pathways and directly interacting with cytoplasmic proteins. In *Bletilla striata* (*B. striata*), lncRNA MSTRG.63923 is suggested to act as a competitive endogenous RNA (ceRNA), competing with BsSWEET1 (a member of the SWEET transporter family) for binding to miR396g/h. This interaction likely modulates polysaccharide accumulation, the primary bioactive components in *B. striata* tubers (Zhang et al. 2023b). In *S. miltiorrhiza*, integrated co-expression and genome localization analyses have uncovered three distinct regulatory modules consisting of four lncRNAs, two mRNAs, and two TFs. These modules offer novel insights into the regulation of diterpenoid biosynthesis (Wang et al. 2023a). Flavonoids are abundant in the leaves of *G. biloba*. In *G. biloba*, bioinformatic analyses have recognized 17 secondary metabolism-related genes as potential targets of 15 lncRNAs, including key enzymes such as PAL and flavonol synthase (FLS) (Wang et al. 2018). Another study in *G. biloba* leaves identified five lncRNAs capable of targeting four genes within the flavonoid biosynthesis pathway (Ye et al. 2019). Li et al. identified two tissue-specific lncRNAs, lnc10 and lnc11, which showed significantly higher expression in immature and mature leaves than in other tissues. Functional validation, achieved through heterologous overexpression in *A. thaliana*, demonstrated that both lncRNAs significantly increased flavonoid accumulation in transgenic lines compared to wild-type controls (Li et al. 2024d). Additionally, Liu et al. described a regulatory module that includes the GbMYB11 and its natural antisense transcript LncNAT11. Their research indicated that GbMYB11 enhances flavonol biosynthesis by upregulating the expression of crucial enzyme genes, flavonoid 3'-hydroxylase (*GbF3’H*) and *GbFLS*. Conversely, LncNAT11 acts as a negative regulator by inhibiting the expression of *GbMYB11* (Liu et al. 2024a). This antagonistic interaction allows precise modulation of flavonoid production in *G. biloba*. In *Hippophae rhamnoides* (sea buckthorn), LNC1 and LNC2 serve as endogenous target mimics (eTMs) for miR156a and miR828a, respectively. Mechanistically, LNC1 suppresses the expression of *SPL9*, whereas LNC2 enhances the expression of *MYB114*, thereby influencing anthocyanin accumulation (Zhang et al. 2018). Additionally, *Malus spectabilis* (*M. spectabilis*) provides insights into ncRNA-mediated regulation of anthocyanin biosynthesis. Under low nitrogen conditions, miR858 downregulates its target, *MsMYB62-like*, through transcript cleavage. Two lncRNAs, eTM858-1 and eTM858-2, serve as eTMs for miR858, mitigating the miR858-mediated cleavage of *MsMYB62-like*, and thus negatively influencing anthocyanin biosynthesis. This regulatory mechanism has been empirically validated in apple fruits (Meng et al. 2023). In tobacco, nta-miRX27 targets *QPT2*, an essential gene in the nicotine biosynthesis pathway that encodes QPT. A lncRNA, nta-eTMX27, functions as an eTM for nta-miRX27, indirectly facilitating nicotine biosynthesis (Xie et al. 2016). By examining two varieties with differing artemisinin contents, three lncRNAs (MSTRG.33718.2, MSTRG.30396.1, and MSTRG.2697.4) associated with key artemisinin biosynthesis genes, including amorpha-4,11-diene synthase (*ADS*), 1-deoxy-D-xylulose-5-phosphate synthase (*DXS*), and 3-hydroxy-3-methylglutaryl-CoA synthase (*HMGS*), were identified (Ma et al. 2024). These findings collectively underscore the significant role of lncRNAs in regulating plant secondary metabolism through the modulation of regulatory and biosynthetic pathway genes.

### Brief introduction to circRNA

circRNAs are a class of single-stranded, covalently closed ncRNA molecules ubiquitously present in eukaryotic organisms (Liu et al. 2022). They are formed by back-splicing, which involves the covalent bonding of the 3’ end to the 5’ end, and they lack both a 5’ cap structure and a 3’ poly(A) tail (Chen 2020). Due to their closed-loop conformation, circRNAs exhibit a higher stability than linear RNAs, which provides resistance to RNase R-mediated degradation. However, the overall abundance of circRNAs is relatively low in vivo, attributed to the intrinsically low efficiency of back-splicing (Sema et al. 2022). circRNAs are widely distributed across chromosomes, as well as within chloroplast and mitochondrial DNA. Depending on their genomic locations, circRNAs can be classified into exonic circRNAs (EcircRNAs), circular intronic RNAs (ciRNAs), exon–intron circRNAs (EIciRNAs), and intergenic circRNAs (Liu et al. 2022). Approximately a decade ago, the presence of circRNAs in plants was first reported in *A. thaliana*. Subsequently, a growing number of circRNAs have been identified in diverse plant groups including cereal crops, oil crops, medicinal plants, and horticultural plants (Li et al. 2024c, Su et al. 2025).

### circRNAs-mediated regulation of SMs biosynthesis

Recent studies on circRNAs in medicinal plants have typically followed a standardized research protocol involving the collection of tissue samples under specified conditions, high-throughput sequencing to identify differentially expressed circRNAs, and bioinformatics analysis to predict potential functions. Jiang et al. established the inaugural systematic catalog of circRNAs in *S. miltiorrhiza* and, notably, predicted that circRNASMscf2473-46,693–46,978 regulates the expression of *SmDXS2* in the tanshinone biosynthetic pathway (Jiang et al. 2021). Furthermore, *S. miltiorrhiza* displays two root color variants, red and white, which differ primarily in tanshinone content. Comparative analysis between these variants revealed 26 differentially expressed circRNAs, suggesting their involvement in tanshinone biosynthesis (Lin et al. 2023). In *Cannabis sativa*, an integrated analysis of transcriptomic and metabolomic data from roots, stems, and leaves identified 10 circRNAs associated with the biosynthesis of six cannabinoids (Liu et al. 2023a). In *H. rhamnoides*, a total of 252 differentially expressed circRNAs were identified across three distinct developmental stages of fruit maturation. Notably, a circRNA (circZDS) derived from the ζ-carotene desaturase (*ZDS*) gene, was predicted to influence carotenoid biosynthesis (Zhang et al. 2019). In *Melastoma candidum*, transcriptome sequencing of flowers at three developmental stages identified three circRNAs, circ000434, circ000302, and circ00012. These circRNAs were predicted to be involved in the biosynthesis of dihydroquercetin, dihydromyricetin, pinobanksin, and *p*-coumaroyl shikimic acid (Li et al. 2023a). In *C. sinensis* (Oolong tea), circRNA498 and circRNA1806 were predicted to be associated with flavonoid biosynthesis (Zhu et al. 2020).

Despite substantial progress in circRNA identification, experimental validation of function remains largely limited. For instance, overexpression of a circRNA derived from phytoene synthase 1 (PSY1) significantly diminished lycopene and β-carotene accumulation in transgenic tomato fruits (Tan et al. 2017). This finding represents one of the few experimentally validations of circRNA-mediated regulation in plant specialized metabolism, offering a crucial reference for functional analyses of circRNAs in medicinal plants. In addition to circRNAs, differentially expressed miRNAs, mRNAs, and other transcripts involved in biosynthetic pathways have also been observed across various tissues or developmental stages in plants. Co‑expression network construction provides a robust approach to elucidate potential regulatory interactions among these RNA species. In *N. tabacum*, regulatory network analysis indicated that miR6024 and miRX282 may regulate nicotine biosynthesis by targeting either mRNAs encoding quinolinate synthase (QS) and aldehyde oxidase 2 (AO2), respectively, or their corresponding circRNAs (circQS and circAO2) (Chen et al. 2019).

### Brief introduction to phasiRNA

In addition to miRNAs, lncRNAs and circRNAs, plants also produce a diverse array of other small RNAs for gene regulation (Fei et al. 2013). Among these, phasiRNAs have emerged as a key class, characterized by their phased biogenesis from precursor transcripts and classified into 21-nt and 24-nt species (Liu et al. 2020). These molecules originate from genomic regions known as PHAS loci, which encode either mRNAs or ncRNAs as precursors. The biogenesis of phasiRNAs is typically initiated by the cleavage of these precursor transcripts by 22-nt miRNAs. Subsequently, the 5’ cleavage fragment is rapidly degraded by a 3’−5’ exonucleolytic complex, while the 3’ fragment is converted into double-stranded RNA (dsRNA) by RNA-dependent RNA polymerase 6 (RDR6), assisted by suppressor of gene silencing 3 (SGS3). The resulting dsRNAs are then processed by Dicer-like (DCL) proteins into 21-nt or 24-nt phasiRNAs (Zhan et al. 2022). Emerging evidence suggests that phasiRNAs play significant biological roles in regulating male reproductive development (Lian et al. 2025), mediating in immune responses (Kong et al. 2022), and adapting to abiotic and biotic stresses (Li et al. 2024e).

### phasiRNAs-mediated regulation of SMs biosynthesis

Mitochondrial transcription termination factors (mTERFs) play essential roles in plant growth, development, and stress responses. In *S. miltiorrhiza*, a specific class of 22-nucleotide miRNAs, termed smi-miRmTERF, cleaves target transcripts including *SmmTERF33* and *SmmTERF45*, thereby initiating phasiRNA biogenesis. Additionally, phasiRNAs cleave *SmmTERF26* transcripts, triggering the production of new phasiRNAs and thereby amplifying the regulatory effects of smi-miRmTERF. Overexpression of smi-miRmTERF significantly enhances the accumulation of phenolic acids, monoterpenes, and sesquiterpenes, suggesting a potential role of phasiRNAs in regulating SMs biosynthesis in *S. miltiorrhiza* (Qiu et al. 2025). In *Actinidia chinensis* (kiwifruit), variations in fruit color — ranging from green and yellow to red and purple — are largely attributable to differential anthocyanin accumulation. Overexpression of *MYB110* enhances anthocyanin production in the fruit flesh. Further research demonstrated that a phasiRNA derived from AcTAS4-D4 (−), generated following miR828-mediated cleavage of *actinidia chinensis transparent testa 4* (*AcTAS4*), directly cleaves *MYB110* transcripts (Wang et al. 2022). Additionally, significant differences in paeoniflorin and flavonoid content have been observed between ornamental and medicinal varieties of *Paeonia lactiflora* (*P. lactiflora*). Comparative analyses identified a phasiRNA Cluster-48340_26405_1_312_ + as a potential regulator of paeoniflorin and flavonoid biosynthesis in the roots (Yuan et al. 2024). In *Glycine max*, the *CHS* genes *CHS1* and *CHS3* produce a long intronic RNA (LIR), which is processed by GmDCL2 to yield primary 22-nt siRNAs. These siRNAs target other *CHS* family members and trigger the biogenesis of secondary 21-nt siRNAs, thereby modulating flavonoid accumulation and affecting seed coat pigmentation (Jia et al. 2020). In *Ipomoea batatas*, mechanical damage induces the expression of sRNA8105. The interaction between sRNA8105 and *IbMYB1* facilitates the generation of secondary siRNAs, which in turn suppress *IbMYB1* expression and promote lignin biosynthesis (Lin et al. 2013).

### ncRNAs-mediated regulation of SMs biosynthesis in other plant species

**Fig. 6:**
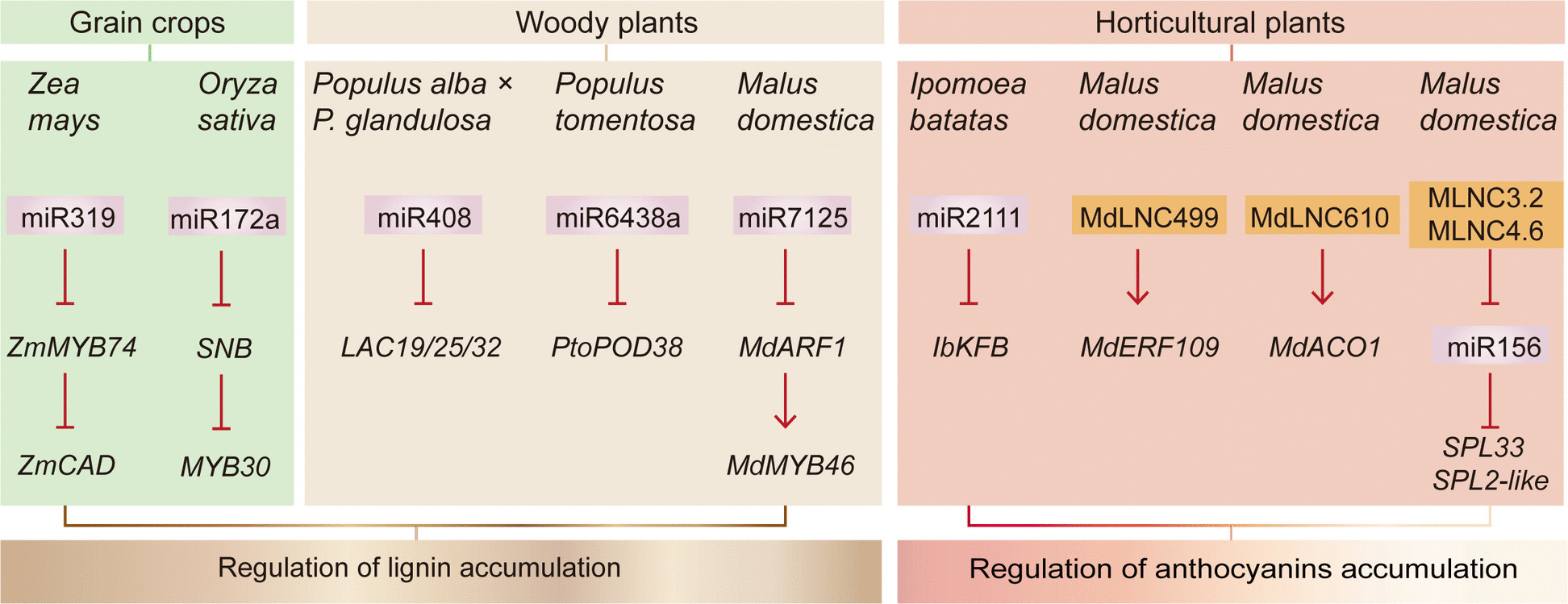
Regulatory roles of ncRNAs in other plant species. The figure illustrates the regulatory roles of ncRNAs in the biosynthesis of related SMs in staple crops, horticultural plants, and woody species. The pink text boxes in the figure represents miRNAs. The orange text boxes represent lncRNAs

To achieve a more comprehensive understanding of ncRNAs in plant metabolism, this review also integrates research findings from additional plant species, including staple crops, horticultural plants, and woody species (Fig. 6).

As principal staple crops, rice and *Zea mays* (maize) produce grains that are rich in starch. However, pests and disease infestations can severely reduce grain yield. Lignin serves as a critical physical barrier that enhances plant resistance against pathogenic invasion. In maize infected with stalk rot disease, the miR319-*ZmMYB74* module promotes lignin accumulation in cell walls by regulating the cinnamyl alcohol dehydrogenase (*ZmCAD*), thereby improving disease resistance (Cao et al. 2025). In rice, pathogen infection induces miR172a expression, which promotes lignin deposition in cell walls and confers broad-spectrum resistance to blast, sheath blight, and bacterial leaf blight (Wang et al. 2024). Additionally, in maize, the miR1432-calmodulin-like protein 21 (*ZmCML21*) module regulates organic acid secretion by modulating the activity of plasma membrane H + -ATPase (ZmPMA2). Suppression of miR1432 enhances organic acid secretion and significantly improves tolerance to low-phosphorus conditions (Pei et al. 2025).

Horticultural plants, including vegetables and fruits, provide both nutritional and ornamental value. In *Prunus persica* (peach) fruits, anthocyanin accumulation is regulated by the expression of *PpMYB10.1*. The miR164a-*PpNACs* module differentially regulates anthocyanin accumulation in peach flesh by modulating *PpMYB10.1* expression (Liang et al. 2025b). In *Lagerstroemia indica*, anthocyanin content in petals increases significantly during floral development from bud to full bloom. LiMYB114 activates anthocyanin biosynthetic genes and exhibits differential expression across different flower color phenotypes. At later stages of petal development, elevated expression of miR828z suppresses *LiMYB114* transcription, thus limiting excessive anthocyanin accumulation (Ni et al. 2025). In *Ipomoea batatas* (sweet potato), the Kelch repeat F-box protein IbKFB negatively regulates anthocyanin biosynthesis. Conversely, miR2111 promotes anthocyanin accumulation in storage roots by suppressing *IbKFB* expression (Tang et al. 2025). Overexpression of the lncRNA MdLNC610 in apple fruits and callus significantly increases 1-aminocyclopropane-1-carboxylate oxidase 1 (*MdACO1*) expression, leading to increased production of both ethylene and anthocyanins (Yu et al. 2022). Moreover, under white or blue light induction, the differentially expressed lncRNAs MLNC3.2 and MLNC4.6 act as eTMs for miR156a, thereby inhibiting miR156a-mediated cleavage of *SPL2-like* and *SPL33* (Yang et al. 2019). Light-induced *MdWRKY1* upregulates the lncRNA MdLNC499, which subsequently enhances the expression of *MdERF109*, promoting biosynthetic gene expression and anthocyanin accumulation (Ma et al. 2021).

Beyond anthocyanin accumulation, ncRNAs also influence fruit flavor and palatability traits. Malic acid content is a key quality determinant of apple fruit acidity. *MdMYB73* activates malate transporter genes, thereby promoting malic acid accumulation. Meanwhile, *MdMYC2* responds to jasmonate (JA) signaling and enhances the expression of miR858. Consequently, miR858 reduces malic acid content by inhibiting *MdMYB73* expression, thus providing a potential target for improving apple flavor (Zhang et al. 2025a). In *Diospyros kaki*, astringency is closely associated with the content of phenolic compounds, particularly tannins. Soluble tannins are converted to insoluble forms in the vacuole via acetaldehyde generated from alcohol dehydrogenase (ADH)-mediated ethanol oxidation. During late fruit development, miR2911a may reduce astringency by regulating *ADH* gene expression (Luo et al. 2015). In addition, the antisense lncRNA of PAL (lncPAL) negatively regulates the biosynthesis of polyphenols in *Siraitia grosvenorii* (Liu et al. 2024c).

Woody plants, including forest trees and perennial crops, have also been extensively studied for ncRNA-mediated regulation of lignin biosynthesis, a major branch of the phenylpropanoid pathway. In *Populus alba* × *Populus glandulosa* (poplar), the auxin (IAA) biosynthesis gene enoyl-CoA hydratase 2 (*PagECH2*) is crucial in the IAA biosynthesis pathway. miR166c directly targets and suppresses *PagECH2* expression, thereby modulating auxin signaling to precisely regulate secondary xylem differentiation and lignin deposition (Zhao et al. 2025a). Additionally, the peroxidase gene *PtoPOD38* in *Populus tomentosa* encodes a lignin polymerization enzyme. Notably, miR6438a negatively regulates *PtoPOD38* expression, thereby inhibiting lignin polymerization and reducing lignin content (Qin et al. 2024). Metabolomic profiling revealed that overexpression of miR156 in poplar leads to an 8.3-fold increase in anthocyanin content, along with increased flavonoid levels and reduced lignin accumulation (Wang et al. 2020). Furthermore, miR408 negatively regulates the expression of *LAC19*, *LAC25*, and *LAC32* in poplar, thereby decreasing lignin polymerization and enhancing cell wall saccharification efficiency, which in turn affects wood quality (Guo et al. 2023).

In addition to developmental regulation, lignin biosynthesis is also modulated by ncRNAs in response to biotic and abiotic stress signals. In apple, the salicylic acid (SA) signaling pathway regulates ncRNA-mediated lignin deposition during pathogen infection. MdARF1 activates *MdMYB46* expression by binding directly to its promoter, thereby promoting lignin accumulation. miR7125 negatively regulates *MdARF1* in response to SA signaling. Upon infection by *Colletotrichum gloeosporioides*, increased SA levels suppress miR7125 expression, leading to enhanced lignin accumulation and improved disease resistance (Liu et al. 2024b). Similarly, in tea plants, growth and quality are also influenced by drought stress, with lignin content closely associated with drought tolerance. Drought stress induces the expression of *CsMYB44* and *CsMYB73*, which suppress *MIR408* expression. Consequently, reduced miR408 levels relieve the repression of its target gene *CsLAC13*, thereby enhancing lignin biosynthesis. Simultaneously, CsMYB44 and CsMYB73 inhibit genes in the flavonoid pathway, reducing catechin accumulation and redirecting metabolic precursors toward lignin synthesis, thus collectively improving drought resistance (Jia et al. 2026).

Collectively, these findings from diverse plant species — ranging from staple crops and horticultural plants to woody species — highlight the functional importance of ncRNA‑mediated regulation across the plant kingdom. These experimentally validated ncRNA-based regulatory modules provide valuable research targets and theoretical foundations for investigating secondary metabolism, growth, development, stress resistance, and quality enhancement in medicinal plants.

## Concluding remarks and future perspective

Over the past few decades, excessive anthropogenic exploitation and ecological degradation have severely threatened the biodiversity of medicinal plants (Huang et al. 2024). A pertinent example is the yew tree, the primary source of the anticancer compound paclitaxel, which is on the verge of extinction (Chen et al. 2016). Concurrently, the global demand for pharmacologically active SMs continues to rise, despite their low natural abundance. Here, we have systematically summarized the regulatory roles of ncRNAs in diverse SM biosynthetic pathways. However, the precise molecular mechanisms through which these ncRNAs exert their regulatory functions remain to be fully elucidated. To advance our understanding of ncRNA-mediated regulatory networks governing SM biosynthesis in medicinal plants, we propose several key directions for future investigation.

The temporal dynamics of ncRNA expression represent a compelling yet relatively underexplored aspect of plant regulatory biology. In *A. annua*, treatment with ABA, MeJA, and SA revealed dynamic expression profiles of miRNAs in response to hormone exposure. For example, following ABA treatment, the expression level of miR156 first increases and then decreases. Under MeJA treatment, miR160 shows an initial decline followed by a subsequent increase, whereas SA treatment leads to a continuous decrease in miR160 expression (Guo et al. 2022b). Furthermore, the temporal expression patterns of ncRNAs influence key transitions in the plant life cycle. In *Rosa chinensis*, the expression level of lncRNA lncWD83 remains relatively low during vegetative growth but increases significantly during reproductive development. lncWD83 promotes flowering by facilitating the ubiquitination and degradation of RcMYC2L, which is mediated by the E3 ubiquitin ligase plant u-box protein 11 (RcPUB11) (Chen et al. 2023). However, most current studies on temporal regulation include only a limited number of time points, potentially missing critical regulatory events that occur at specific junctures. Single-cell RNA sequencing (scRNA-seq) is a powerful method that enables high-resolution temporal dynamic analysis. Using this approach, a high-resolution cell atlas of *A. annua* was constructed, revealing that the six secretory cells within glandular trichomes are the sites of artemisinin biosynthesis. In addition, pseudotime analysis reconstructed the developmental trajectory of epidermal cells as they differentiate into glandular trichomes and T-shaped non-glandular trichomes, revealing that glandular trichome development is accompanied by fatty acid metabolism and terpenoid biosynthesis (Zhang et al. 2025b). Thus, scRNA-seq not only reconstructs developmental trajectories of cellular lineages but also represents a promising approach for elucidating the ncRNAs-mediated temporal regulatory network in medicinal plants.

Given that SMs accumulation in medicinal plants often exhibits pronounced spatial specificity, the transport and distribution of ncRNAs in the spatial dimension also warrant in-depth investigation. For example, in *S. miltiorrhiza*, tanshinones accumulate specifically in the root periderm. Notably, integrated multi-omics analysis of early root development has revealed spatiotemporal regulatory networks, in which TFs such as ERF105 play dual roles in coordinately regulating both tanshinone biosynthesis and transport (Li et al. 2026). In addition, ncRNAs themselves can display spatial specificity through tissue‑specific synthesis and intercellular transport. In *Lotus japonicus*, miR2111 is synthesized exclusively in the leaf phloem and then transported over long distances to the roots. There, it suppresses the autoregulation of the nodulation system, thereby maintaining susceptibility to rhizobia and promoting the formation of nitrogen-fixing nodules (Tsikou et al. 2018). Therefore, to further advance our understanding, multi‑omics spatiotemporal technologies that simultaneously detect metabolites, ncRNAs, and mRNAs can be employed to construct spatiotemporal regulatory networks. Such approaches would help elucidate the mechanisms by which plant ncRNAs achieve spatial transport and maintain the dynamic equilibrium of their regulatory functions.

**Fig. 7:**
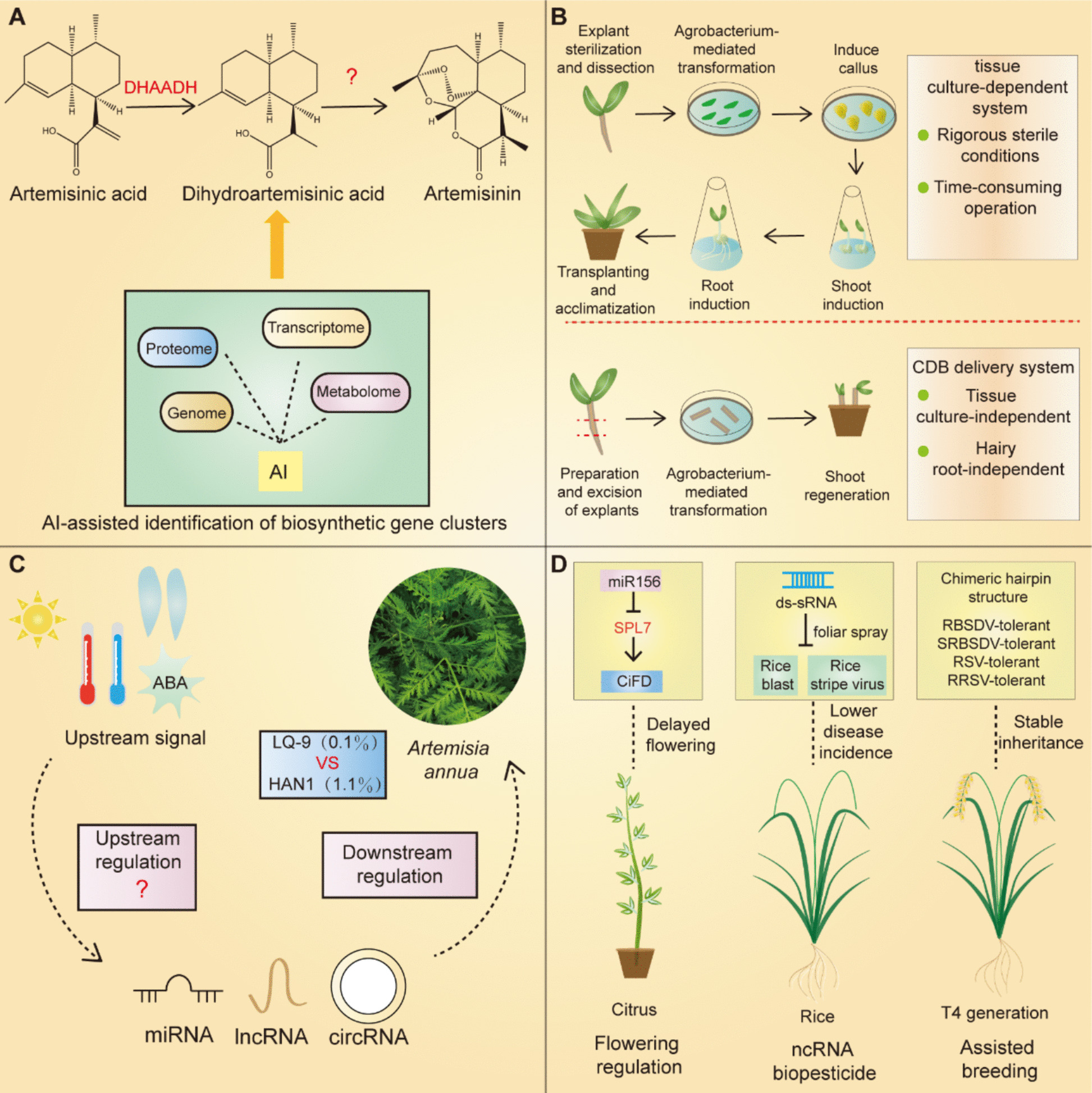
Future research prospects for ncRNAs in medicinal plants. **A**, elucidation of biosynthetic pathways and AI-assisted efficiency enhancement. Effective regulation of SM biosynthesis requires comprehensive elucidation of the underlying metabolic pathways. AI offers powerful tools for rapidly integrating multi-omics data, thereby accelerating pathway discovery and enhancing research efficiency. **B**. Optimization of plant genetic transformation systems. Conventional genetic transformation methods relying on plant tissue culture require sterile conditions and are often time-consuming. The operation process of the CDB delivery system is relatively simple and convenient. **C**. Upstream regulatory mechanisms of ncRNAs. Investigating how upstream signals such as temperature, light, and water affect t ncRNAs expression can help explain the resulting differences in SM accumulation levels. **D**. Application strategies for ncRNA. The regulatory capacity of ncRNAs holds substantial promise for practical applications in medicinal plant production. RBSDV, rice black-streaked dwarf virus; SRBSDV, southern rice black-streaked dwarf virus; RSV, rice stripe virus; RRSV, rice ragged stunt virus

The elucidation of biosynthetic pathways forms the foundation for understanding the regulatory networks that govern secondary metabolism in medicinal plants. Within these pathways, the expression of key enzyme genes acts as “road signs” that direct metabolic flux and determine the trajectory of subsequent biochemical transformations. Paclitaxel, a widely used anticancer agent, has long attracted significant attention due to the complexity of its biosynthetic pathway. Baccatin III serves as a central precursor in paclitaxel biosynthesis. Through integrated multi-omics analysis and functional validation, two novel biosynthetic enzymes, taxane oxetanase (TOT) and taxane 9α-hydroxylase (T9αH), were identified, enabling the successful heterologous biosynthesis of baccatin III in tobacco (Jiang et al. 2024a). Subsequently, the discovery of two key modifying enzymes, 2-oxoglutarate-dependent dioxygenase (T2’OGD) and taxoid-3’-N-benzoyl-transferase (T3’NBT), involved in the conversion of baccatin III to paclitaxel, has filled the last remaining gap in the paclitaxel biosynthetic pathway (Liang et al. 2025a). These findings collectively establish a solid foundation for understanding and manipulating paclitaxel biosynthesis in plants. However, the natural product biosynthetic pathways of most medicinal plants remain largely unelucidated. In *A. annua*, the conversion of artemisinic acid (AA) to artemisinin has long been a research focus. The most plausible pathway currently involves the sequential transformation of AA to dihydroartemisinic acid (DHAA), followed by the conversion of DHAA to artemisinin. The recent discovery of dihydroartemisinic acid dehydrogenase (DHAADH), which catalyzes the conversion of AA to DHAA, has bridged a critical gap (Guo et al. 2025). Nevertheless, the specific mechanism underlying the formation of the peroxy bridge during the conversion of DHAA to artemisinin remains a key unresolved question. In particular, whether this transformation is enzymatic or a light-induced non-enzymatic photochemical process continues to be debated (Xu et al. 2022). These pathway gaps significantly hinder investigations into regulatory mechanisms. Therefore, comprehensive elucidation of SMs biosynthetic pathways should be an essential prerequisite for subsequent regulatory studies (Fig. 7A).

In recent years, the rapid advancement of artificial intelligence (AI) technologies has provided powerful analytical tools for research in plant biology. AI offers significant potential for predicting and prioritizing miRNA targeted genes. One example is miRNA Chaos Game Representation (miCGR), an end-to-end deep learning framework, which predicts functional miRNA targets using enhanced CGR. Unlike traditional sequence feature-based methods, miCGR converts gene sequences into two-dimensional graphics, thereby outperforming existing methods (Wu et al. 2024). Furthermore, AI can be leveraged to substantially enhance gene editing efficiency. The tRNA-processing system enhances the efficiency of CRISPR-Cas-based multiplex genome editing in plants. However, conventional algorithms for tRNA prediction show poor sensitivity when identifying noncanonical tRNAs. Based on the Mamba architecture and 14 plant genome datasets, plant tRNA large language models (tLLMs) were developed. tLLMs serve as a robust platform for tRNA-based CRISPR-Cas expression and offer a comprehensive tRNA resource for plant genome editing (He et al. 2026). Identification of candidate genes to be edited usually requires genome-wide screening. However, this approach often yields numerous candidate genes, greatly complicating the identification of truly relevant ones. To address this challenge, MedMeta, a comprehensive database, can be leveraged. MedMeta integrates SMs with genomic, biochemical, and pharmacological information from thousands of medicinal plants. Through AI-assisted functional annotation, MedMeta links SMs to the genomic data, thereby facilitating the exploration of biosynthetic pathways and regulatory networks (Meng et al. 2025). Therefore, AI-powered multi-omics integration — encompassing plant phenotypic data, ncRNAs, mRNAs, proteins, and metabolites — will significantly accelerate the elucidation of signaling pathways and the identification of high-confidence regulatory targets.

Nonetheless, candidate genes identified by AI algorithms or other approaches require further validation to confirm their biological functions in vivo. Currently, apart from a few well-studied medicinal plants such as *S. miltiorrhiza* and *A. annua*, the biological roles of ncRNAs and their target genes remain unverified in most medicinal plants. Therefore, establishing efficient and stable genetic transformation systems is a priority for future research. Notably, the cut-dip-budding (CDB) gene delivery system bypasses traditional tissue culture and enables high-efficiency delivery of gene editing vectors. It has been successfully applied to *Taraxacum mongolicum*, *Rehmannia glutinosa*, *S. miltiorrhiza*, and other medicinal plants. The CDB method capitalizes on the exceptional regenerative capacity of explants, significantly improving the transformation efficiency, particularly for medicinal and other less-studied plants (Cao et al. 2024a). Further optimization of gene editing tools can also greatly advance functional studies of ncRNAs and their target genes. For example, CRISPR-Cas9 enables precise knockout, insertion, or replacement of candidate genes. A promising strategy involves substituting commonly used exogenous 35S promoters with endogenous alternatives. In *S. miltiorrhiza*, a tRNA-gRNA system driven by the endogenous ribosomal protein S5 A (SmRPS5A) promoter enables simultaneous multiplex gene editing, thereby surpassing conventional hairy root- and protoplast-based methods (Zheng et al. 2025). Therefore, the continued development of efficient and stable genetic transformation systems will substantially advance research into ncRNA-mediated regulation of secondary metabolism in medicinal plants (Fig. 7B).

Current research predominantly focuses on how ncRNAs regulate SMs biosynthesis in medicinal plants, particularly examining downstream biosynthetic pathways. However, the upstream regulatory mechanisms — how plants perceive external signals and regulate ncRNA expression — remain comparatively underexplored. Addressing this knowledge gap is crucial for understanding “Daodi”, which refers to the superior quality and clinical efficacy of medicinal materials originating from specific geographic regions with optimal ecological conditions. A significant variation in SMs content is a distinguishing characteristic of “Daodi” properties, suggesting that diverse regional environmental conditions may foster the development of these unique chemotypes. For example, the *A. annua* cultivar HAN1, originating from southern China, exhibits an artemisinin content of 1.1%, in contrast to LQ-9 from northern China, which contains only 0.1% (Liao et al. 2022). Whether ncRNAs are involved in the differential accumulation of artemisinin between two cultivars remains unknown. Understanding how external signals affects SMs biosynthesis via ncRNA expression could provide valuable insights into the molecular basis of the “Daodi” characteristics (Fig. 7C). In addition to upstream regulatory mechanisms, species-specific ncRNA studies also warrant attention. For example, in *S. miltiorrhiza*, the species-specific smi-miRmTERF regulates the expression of 18 unique SmTERF genes, promoting SM accumulation and stress resistance (Qiu et al. 2025). Therefore, beyond conserved miRNAs like miR156 and miR160, species-specific miRNAs may offer key insight for specific SM research.

The potential applications of ncRNAs in improving the production of medicinal plants also merit careful consideration (Fig. 7D). For medicinal plants like *Carthamus tinctorius* and *Lonicera japonica* (*L. japonica*), the flowering period is a critical phenological stage for harvesting medicinal organs. Precise control of flowering time can significantly enhance economic returns. miR156 is widely recognized as a key regulator of the transition from vegetative to reproductive growth in plants (Yang et al. 2023). In citrus, the flowering gene bZIP transcription factor FD (*CiFD*) is activated by *CiSPL7*, a target of miR156c. However, miR156c inhibits the activation of *CiFD* by targeting and suppressing *CiSPL7* expression, thereby delaying the flowering (Chen et al. 2025b). In the future, the identification of flowering-related ncRNAs in medicinal plants could help optimize the balance between vegetative and reproductive growth by enabling precise manipulation of flowering time.

RNA interference (RNAi) technology has received significant attention for its potential in developing biopesticides. These RNAi-based agents can specifically target and silence essential genes in pathogens or pests, thereby enabling precise management of plant diseases and insect pests (Yue et al. 2025). During the growth of rice, the crop may be attacked by pathogens such as the rice blast virus and rice stripe virus. The spray application of dsRNA effectively penetrates the cell membranes of *Magnaporthe oryzae*. This induces gene silencing and inhibits conidia germination and appressorium formation. Furthermore, layered double hydroxide (LDH) nanomaterials significantly enhance the stability and delivery efficiency of RNA, achieving long-term protection for rice (Chen et al. 2025c). Medicinal plants are subject to diverse pest and disease pressures, and the control efficacy of single chemical pesticides is often limited. Consequently, the high precision offered by RNAi technology holds substantial potential for application. Designing nanomaterials to improve RNA loading and delivery efficiency represents a promising direction for advancing its application in medicinal plant cultivation.

Medicinal plants exhibit considerable genetic diversity, which leads to significant variation in the content of bioactive components among different varieties. Cultivating high-yield, high-resistance elite varieties represents a crucial objective for the standardized production of medicinal plants. The ncRNA-mediated regulation of developmental traits provides a highly promising tool for breeding applications. Plants with ncRNA overexpression or silencing could be generated by precisely modifying or editing ncRNA precursor sequences, thereby providing essential resources for the development of elite varieties. Research in rice offers a practical reference for related studies in medicinal plants. For example, by analyzing the key protein-coding sequences of four rice viruses, 300 bp conserved fragments were cloned from each and assembled into a single hairpin RNA structure. This fusion hairpin was introduced into rice callus tissues via *Agrobacterium*-mediated transformation, ultimately generating the stable transgenic rice line ZJU-4 K. This line constitutively produces abundant siRNAs that specifically target conserved sequences of the four viral gene (Li et al. 2024b). Therefore, developing ncRNAs as novel molecular markers associated with desirable phenotypes could substantially enhance breeding efficiency and advance the standardized production of medicinal plants.

## Data Availability

Not applicable.

## Abbreviations

AbfA: Alpha-L-arabinofuranosidase
ADH: Alcohol dehydrogenase
ADS: Amorpha-4,11-diene synthase
ANS: Anthocyanidin synthase
BIA: Benzylisoquinoline alkaloids
CCR: Cinnamoyl-CoA reductase
CHI: Chalcone isomerase
CHS: Chalcone synthase
CPS1: Copalyl diphosphate synthase 1
DFR: Dihydroflavonol 4-Reductase
DHAA: Dihydroartemisinic acid
DHAADH: Dihydroartemisinic acid dehydrogenase
DXS: 1-Deoxy-D-xylulose-5-phosphate synthase
ERF84: Ethylene response factor 84
ETM: Endogenous target mimic
FLS: Flavonol synthase
FNS II: Flavone synthase II
F3H: Flavanone 3-hydroxylase
G10H: Geraniol 10-hydroxylase
HEN1: Hua enhancer 1
HMGR: Hydroxymethylglutaryl-CoA reductase
HMGS: 3-Hydroxy-3-methylglutaryl-CoA synthase
HY5: Elongated hypocotyl 5
KSL1: Kaurene synthase-like 1
LAC: Laccase
PAL: Phenylalanine ammonia-lyase
PSY1: Phytoene synthase 1
RISC: RNA-induced silencing complex
TAT: Tyrosine aminotransferase
TDC: Tryptophan decarboxylase
ZDS: ζ-Carotene desaturase
